# Determination of diclofenac concentrations in human plasma using a sensitive gas chromatography mass spectrometry method

**DOI:** 10.1186/s13065-016-0199-3

**Published:** 2016-08-17

**Authors:** Iltaf Shah, James Barker, Declan P. Naughton, Stephen J. Barton, Syed Salman Ashraf

**Affiliations:** 1 School of Life Sciences, Pharmacy and Chemistry, Kingston University, Penrhyn Road, Kingston-upon-Thames, Surrey, KT1 2EE UK; 2 Department of Chemistry, College of Science, United Arab Emirates University, Al Ain, UAE

**Keywords:** Human plasma, Diclofenac sodium, GCMS, Sensitive GCMS

## Abstract

**Background:**

A gas chromatography mass spectrometry (GCMS) method for the determination of diclofenac in human plasma has been developed and validated.

**Results:**

This method utilizes hexane which is a relatively less toxic extraction solvent compared to heptane and benzene. In addition, phosphoric acid and acetone were added to the samples as deproteination agents, which increased the recovery of diclofenac. These revised processes allow clean extraction and near-quantitative recovery of analyte (approx. 89–95 %). Separation was achieved on a BP-1 column with helium as carrier gas. The molecular ion peaks of the indolinone derivatives of diclofenac ion (m/z 277) and the internal standard, 4-hydroxydiclofenac ion (m/z 439) were monitored by a mass-selective detector using selected ion monitoring (SIM) mode. The linear range for the newly developed and highly sensitive assay was between 0.25–50 ng/mL. The detection and lower quantifiable limits were 0.125 and 0.25 ng/mL, respectively. The inter-day and intra-day coefficients of variation for high, medium and low quality control concentrations were less than 9 %. The robustness and efficacy of this sensitive GCMS method was further demonstrated by using it for a pharmacokinetic study of an oral dosage form of diclofenac, 100 mg of modified-release capsules (Rhumalgan XL), in human plasma.

**Conclusions:**

This method is rapid, sensitive, specific, reproducible and robust, and offers improved sensitivity over previous methods. This method has considerable potential to be used for detailed pharmacokinetics, pharmacodynamics and bioequivalence studies of diclofenac in humans.

## Background

Sodium 2-(2,6-dichlorophenyl-aminophenyl acetate (diclofenac) salt is a nonsteroidal anti-inflammatory drug. Clinically, it is mostly used for the treatment of pain caused by inflammation [1–6]. In humans, “the absorption, distribution, metabolism, and excretion” (ADME) studies of diclofenac show that it has high inter- and intra-subject variability [7–17] that may arise from pharmacogenomics differences amongst individuals and/or precision in measurement procedures. Furthermore, due to wide availability of diclofenac formulations, there is an interest in having robust and sensitive assays for diclofenac quantitation for pharmacokinetics studies. This could be especially important in developing countries with nascent pharmaceutical industries, who may synthesize and sell their own diclofenac formulations. To start this inquest, we have developed a gas chromatographic mass spectrometric (GCMS) method for the detection and quantification of diclofenac concentrations in human plasma. Many methods have been developed for the determination of diclofenac in biological specimens e.g. high pressure liquid chromatography with ultra violet detection (HPLC–UV) [18–21], HPLC with electrochemical detection [22, 23] and online micro-dialysis with liquid chromatography [24], electro-membrane extraction (EME) and pulsed- electro-membrane extraction (PEME) coupled with HPLC [25], liquid chromatography mass spectrometry (LCMS) [26, 27] and GCMS methods [28–31].

While GCMS methods have been the favorite choice in the past, many derivatisation reagents have been tried and tested. Borenstein et al. used pentafluoropropionic anhydride (PFPA) as a derivatising agent with lower limit of quantification (LOQ) of 1 ng/mL with a 95 % recovery [30]. Choi et al. used a mixture of PFPA and a mixture (1000:2:3,v/w/w) of *N*-methyl-*N*-trimethylsilyltrifluoroacetamide (MSTFA), ammonium iodide (NH_4_I), and dithioerythritol (DTE) as derivatisation reagent. With this method the LOQ was 0.5 ng/mL and the recovery approximately 97 % [32]. Yilmaz et al. described a method where MSTFA was used as the derivatising agent (silylating reagent), and the hydroxyl group of diclofenac was O-silylated. Here the LOQ was 5 ng/mL with a recovery of about 96 % [31]. In our work PFPA was chosen as the best derivatising agent due to it giving a better sensitivity and maximum recovery.

HPLC-UV methods have been reported to measure plasma diclofenac in the range *ca.* 10–100 ng/mL [18–20]. Plasma matrix and other diclofenac metabolites are also known to cause interferences in accurate diclofenac estimation in human matrices [29]. To ensure good specificity and reproducibility, lengthy and comprehensive sample preparation procedures are often required [16–18]. On the other hand, mass spectrometric methods offer potentially better precision, accuracy, sensitivity and recovery, with a detection limit of between 0.2–2 ng/mL [26, 27, 30, 33]. The reported mass spectrometric methods used benzene and heptane as extraction solvents. However, the sensitivity of these methods was not good enough to carry out a thorough and accurate lower dose pharmacokinetic analysis of diclofenac in human plasma. In the present study, we have modified existing methods [29–31] introducing hexane, acetone and sodium bicarbonate to develop a more sensitive, specific and reproducible method for the determination of diclofenac in human plasma.

Having developed and validated a method for the quantification of diclofenac in plasma we sought to demonstrate a proof-of-concept application. For this purpose, plasma samples were obtained from 30 volunteers who had been given an oral dosage of 100 mg of diclofenac sodium (Rhumalgan XL 100 mg modified-release capsules). Human plasma samples were analysed between 0 and 12 h to evaluate the pharmacokinetic parameters of diclofenac.

## Methods

### Chemicals and reagents

Diclofenac sodium salt (analytical standard), 4-hydroxydichlofenac (>98 % pure), concentrated phosphoric acid solution of 85 % (w/v), derivatising agent PFPA (99 % pure) and sodium hydrogen carbonate (>99.7 % pure) were purchased from Sigma-Aldrich Ltd Dorset, UK. Methanol (MeOH), acetone, chloroform, water and hexane of HPLC grade were purchased from Hichrom Ltd, Reading, Berks, UK. Drug free human plasma was obtained from TCS Biosciences Ltd, Buckingham, UK.

### Apparatus and assay conditions

GCMS was performed with a Hewlett Packard model 6890 Gas Chromatograph (GC) fitted with a 6890 autoinjector for a pulsed splitless injection coupled to a model 5973 Mass Selective Detector (MSD) (Agilent Technologies, USA). Separation was achieved using a BP-1 fused silica capillary column (15 m × 250 µm × 0.25 µm). Helium (99.95 %, BOC Gases, Surrey, UK) was used as a carrier gas at a flow-rate of 1.2 mL/min. The injection volume was 2 µL. The syringe size was 10 µL. Pulse pressure and pulse time were 20 psi and 0.5 min respectively. Total run time was 14.5 min. Injector temperature was 280 °C. The initial oven temperature was 150 °C, whilst the final oven temperature was 300 °C. The final high temperature purged residual materials from the column. The column temperature was initially held at 150 °C for 4 min (total run time 4 min), increased at 4 °C/min to 180 °C in 7.5 min and held there for 0.5 min (run time 12 min), then increased at 60 °C/min to 300 °C in 2 min and held there for 0.5 min (run time 14.5 min). Carrier gas flow-rate at the split vent was 54.3 mL/min. The injector was set to auto clean itself by pre-injecting hexane.

The mass selective detector was operated in the selected ion monitoring mode (with electron impact) and set at m/z [M^+^] 214, 242 and 277 and m/z 376 and 439 for the detection of diclofenac and 4-hydroxydiclofenac, respectively. The corresponding retention times of diclofenac and 4-hydroxydiclofenac were 7.5 min and 8.5 min respectively (for a 100 ms dwell). The relative retention times of diclofenac to 4-hydroxydiclofenac was 1.13 with a standard deviation of 0.01. Solvent delay was 3 min, electron multiplier accelerating voltage 2494 V and electron ionisation energy 70 eV. Mass spectrometer source, quadrupole and transfer line temperatures were 230, 150 and 280 °C, respectively. The accelerating voltage was set at 3.5 kV. The system was controlled and detector output data was processed using a Chemstation version B.00.02 software.

### Preparation of standards

A stock solution of 1 mg/mL was prepared by adding 10.78 mg of Diclofenac in 10 mL (MeOH) (Final conc. 1.078 mg/mL). Working solutions were prepared by serial dilutions of the stock solution. A 0.45 mg/mL stock solution of 4-hydroxydiclofenac was prepared by dissolving 4.5 mg of 4-hydroxydiclofenac in 10 mL MeOH. The concentration of the working internal standard solution of 4-hydroxydiclofenac was 0.0045 mg/mL. All solutions were stored at −20 °C.

#### Preparation of 1M phosphoric acid

33.3 mL of concentrated phosphoric acid solution of 85 % (w/v) strength was diluted with 500 mL deionised water to give a solution of 1 M concentration. The bottle was labelled and an expiry date of 2 months from the date of preparation was applied. The solutions were found to be stable for this duration. The solution was stored at room temperature.

#### Preparation of 0.08 M sodium hydrogen-carbonate solution

Approximately 0.672 g of sodium hydrogen carbonate was weighed and diluted with 100 mL of HPLC grade water, stored at room temperature with an expiry date of 2 months.

### Sample preparation

Appropriately labelled Pyrex glass tubes (100 × 13 mm) with screw caps were used. Plasma samples (1 mL) were added to the sample tubes. An internal standard of 4-hydroxydiclofenac (25 μL) of concentration 0.0045 mg/mL was added and the mixtures acidified and vortex mixed with1 M phosphoric acid (1 mL). Then, to all tubes, 1 mL of acetone was added for deproteination followed by vortex mixing. Next, 5 mL of n-hexane was added, the tubes capped and the samples placed on a roller mixer for 15 min. All the tubes were centrifuged at 1400×*g* for 5 min at room temperature. The top hexane layer was transferred to glass screw-capped tubes to which 1 mL of 0.08 M sodium hydrogen carbonate solution was added for basification and to increase partition of the drug into the aqueous layer. The tubes were capped and again placed on a roller mixer for 15 min and centrifuged at 3000×*g* for 5 min. The upper hexane layer was aspirated and discarded. Phosphoric acid (1 mL) was then added, followed by 5 mL of n-hexane. The tubes were then placed on a roller mixture for a further 15 min and centrifuged for 5 min at 3000×*g* and the top hexane layer was transferred to glass tubes (100 × 13 mm, without screw cap). Hexane was then evaporated off under a stream of nitrogen with the heater block set at 35 °C.

### Derivitisation of the samples

**Fig. 1:**
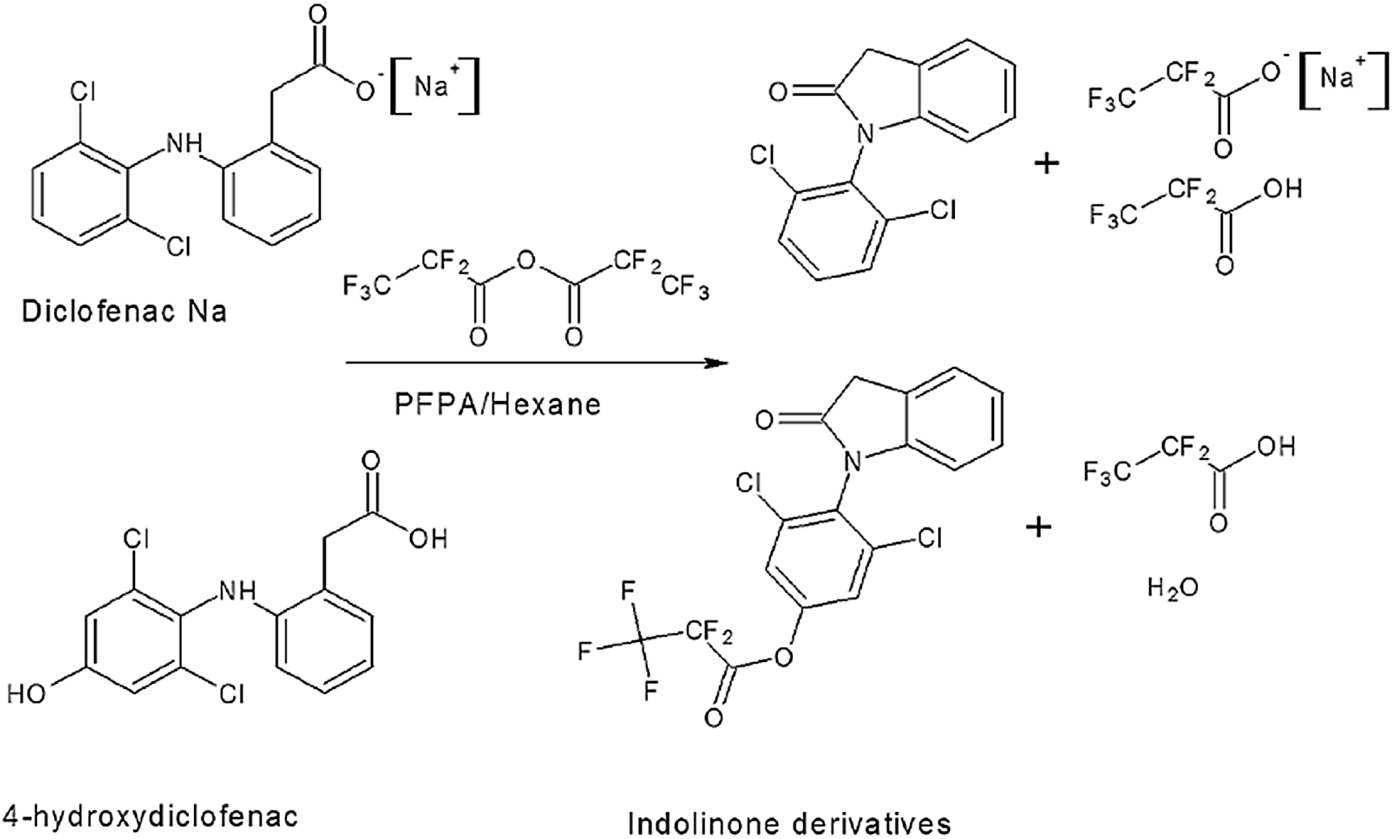
Formation of indolinone derivatives for both diclofenac-Na and 4-hydroxydiclofenac in the presence of derivatising agent PFPA

n-Hexane (975 μL) and 25 μL (v/v) of PFPA were added to the dried residue and the tubes vortex mixed for 30 s. The samples were allowed to react for 30 min on a heater block at 35 °C and gently evaporated under a stream of nitrogen. The tubes were allowed to cool to room temperature and the derivatised compound was reconstituted into 80 μL of chloroform. The sample was transferred to autosampler vials and the GCMS autosampler programmed to inject 2 μL of the sample. Figure 1 shows the indolinone derivatives formed from derivatisation of diclofenac sodium and 4-hydroxydiclofenac using the derivatising agent PFPA.

This newly developed analytical method was tested in a human pharmacokinetic study. Plasma samples obtained after administration of 100 mg of oral diclofenac sodium in participating volunteers were analysed to quantitate the plasma concentrations of the drug over a 12 h period. Kingston University research ethics committee approved the protocol and the volunteers provided informed written consent to participate.

## Validation

### Calibration curve and analysis

The working standard solutions for plasma analysis were made by serial dilution of the stock solutions to final concentrations of 10, 20, 40, 200, 400, 1000 and 2000 ng/mL in methanol. Calibration standards were obtained by spiking 25 µL of each of these standards into 975 µL of human plasma to produce concentrations of 0.25, 0.5, 1, 5, 10, 25 and 50 ng/mL. The samples for the standard curve were processed as described in the materials and method section. The ratio of peak area of diclofenac to that of the internal standard was plotted versus the concentration of the diclofenac in the calibration standard and a least-squares linear regression analysis was performed. Values of unknown plasma concentrations were determined from the regression line of this calibration curve. The working quality control solutions in methanol for plasma analysis were made by serial dilution of the stock solutions to obtain final concentrations of 10, 20, 44, 600 and 1600 ng/mL. Quality controls were obtained by spiking 25 µL of each of these standards into 975 µL of human plasma to produce concentrations of 0.25, 0.5, 1.1, 15 and 40 ng/mL. All methanolic solutions were stored at 2–8 °C with an expiry of 7 days, due to their short stability in methanol, while plasma samples were stored at −20 °C.

### Intra–inter day precision and accuracy

The accuracy and precision of the method was determined by assaying 0.5 mL aliquots of ethylene-diamine-tetra-acetic acid (EDTA) human plasma fortified with four quality control (QC) samples of 0.5, 1.1, 15.0 and 40.0 ng/mL of diclofenac. These fortified samples were later assayed by GCMS. To assess the inter-assay precision and accuracy, samples were analysed on five separate days. To assess the intra-assay precision, these same QC concentrations were analysed and compared during 1 day.

### Linearity, sensitivity and specificity

The ratio of diclofenac and 4-hydroxydiclofenac responses were plotted by GCMS ChemStation Version 3.1 software to determine the linearity. A calibration point was rejected as an outlier if the back-calculated concentration for a calibrator (on the basis of the corresponding calibration curve) deviated by more than 15 % at all concentrations covered by the calibration range, except at the lower limit of quantitation (LLOQ), where a deviation of 20 % was acceptable. A calibration curve was allowed with a minimum of four acceptable calibration levels. These criteria were based on the US Food and Drug Administration (FDA) “Bioanalytical Method Validation: Guidance for Industry” protocol [34].

The analytical method was able to determine diclofenac and 4-hydroxydiclofenac (internal standard) in plasma without significant interference from other endogenous compounds. The specificity of the validated assay procedure was shown by analysing 6 blank plasma samples from subjects not exposed to diclofenac, it was then spiked and recoveries calculated.

### Extraction recovery

Absolute extraction recovery of diclofenac from human EDTA plasma was determined at three concentration levels: 1.1, 15 and 40 ng/mL. The area ratio response of diclofenac to internal standard in the extracted sample divided by the area ratio response determined in an un-extracted sample and multiplied by 100 gave the percent recovery. These samples were extracted, as described earlier, except that the internal standard was added to the collected extract. The concentrations of the spiked plasma samples were calculated from the curve and compared to the theoretical values in order to calculate the extraction recovery.

### Stability

The stability of diclofenac in human EDTA plasma was determined in processed sample extracts over at least 24 h period and also by three repeated freezing and thawing cycles.

### Stability of diclofenac in EDTA plasma to repeated freezing and thawing cycles

Human EDTA plasma samples at concentration of QCL = 1.1 ng/mL, QCM = 15 ng/mL and QCH = 40 ng/mL were subjected to three freezing and thawing cycles. The time span for freeze/thaw cycles was 72 h with each freeze/thaw cycle lasting for 24 h with time points 24, 48 and 72 h. The results obtained after each freezing and thawing cycle were expressed as a percentage change from the results for QCL = 1.1 ng/mL, QCM = 15 ng/mL and QCH = 40 ng/mL in the intra-assay run (validation run-1, these samples were prepared fresh and had not experienced any freezing conditions). The test compound was considered to be stable if the percentage change from freshly prepared samples was within ±15 % of the nominally spiked level.

### Pharmacokinetics study

The pharmacokinetic study chosen, set out to analyze diclofenac sodium in human plasma. For this study, plasma samples were obtained from 30 volunteers who had been given an oral dosage of 100 mg of diclofenac sodium (Rhumalgan XL™ 100 mg modified-release capsules). Diclofenac concentrations in plasma were measured between 0 and 12 h, (blood being collected every hour) in order to evaluate the pharmacokinetic parameters of diclofenac. Kingston University Faculty of Science Research Ethics Committee approved the protocol and the volunteers provided informed written consent to participate. The pharmacokinetic study was conducted according to the principles of the Declaration of Helsinki [35]. According to FDA guidelines for generic drugs studies, the area under the curve (AUC) was calculated using a linear trapezoidal method, by applying non-compartmental data analysis. The method developed was used to investigate the plasma profile after oral dosing of diclofenac sodium 100 mg capsules in 30 healthy young male volunteers.

## Results and discussion

Diclofenac sodium and 4-hydroxydiclofenac react with the derivatising agent PFPA to form indolinone derivatives, which upon electron ionisation gave rise to diclofenac ions at m/z 277, 242 and 214, whilst 4-hydroxydiclofenac gave ions at m/z 439 and 376.

### Calibration curve and analysis

**Fig. 2:**
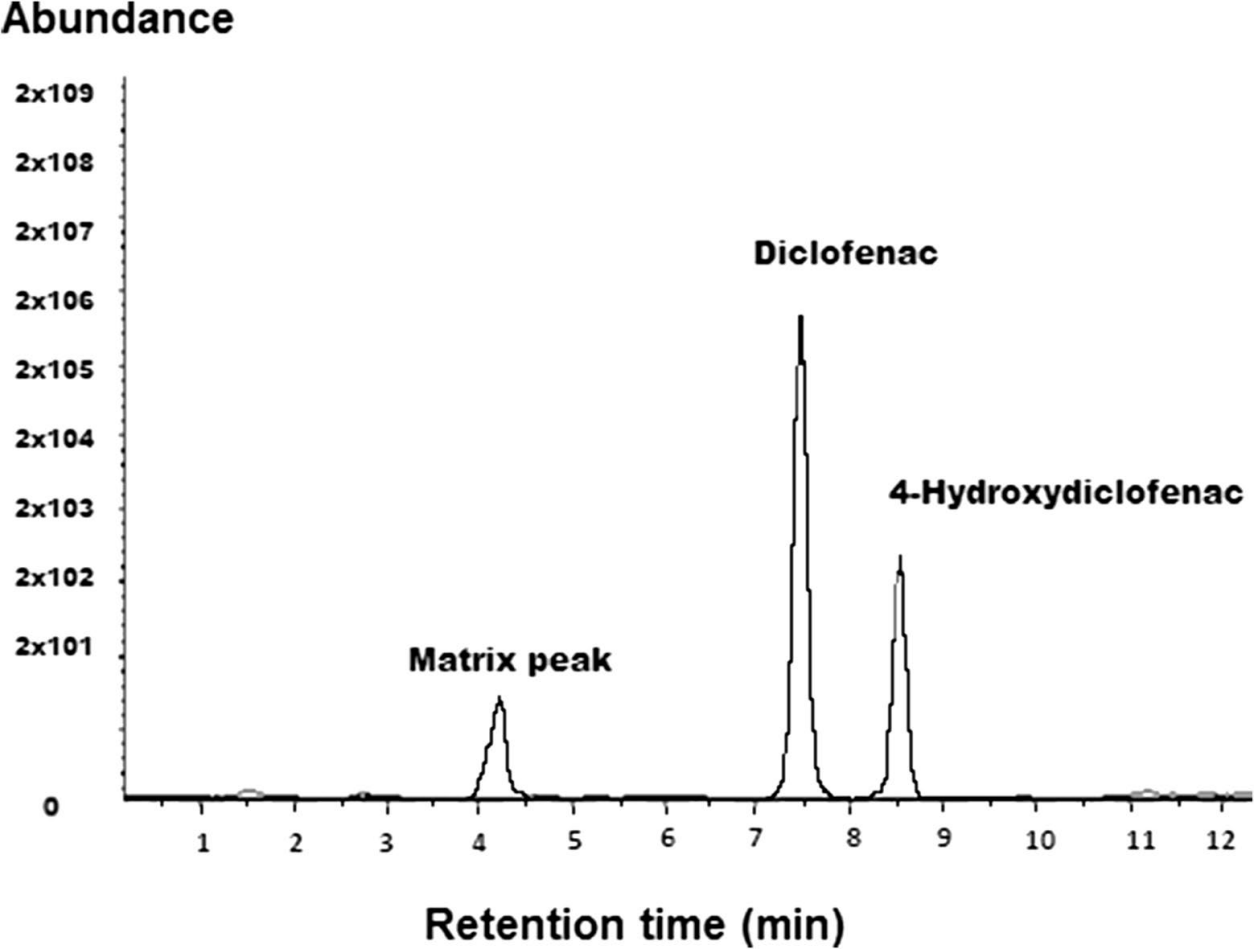
Chromatographs of diclofenac (0.25 ng/mL) and 4-hydroxydiclofenac (0.0045 ng/mL) derivatives in plasma

Figure 2 displays a representative chromatogram of blank plasma spiked with 0.25 ng/mL of diclofenac and 0.0045 ng/mL of the internal standard. Pooled normal human plasma yielded relatively clean chromatograms with no significant interfering peaks. Both diclofenac and the internal standard showed sharp, well-defined peaks at retention times of 7.5 and 8.5 min, respectively.

**Fig. 3:**
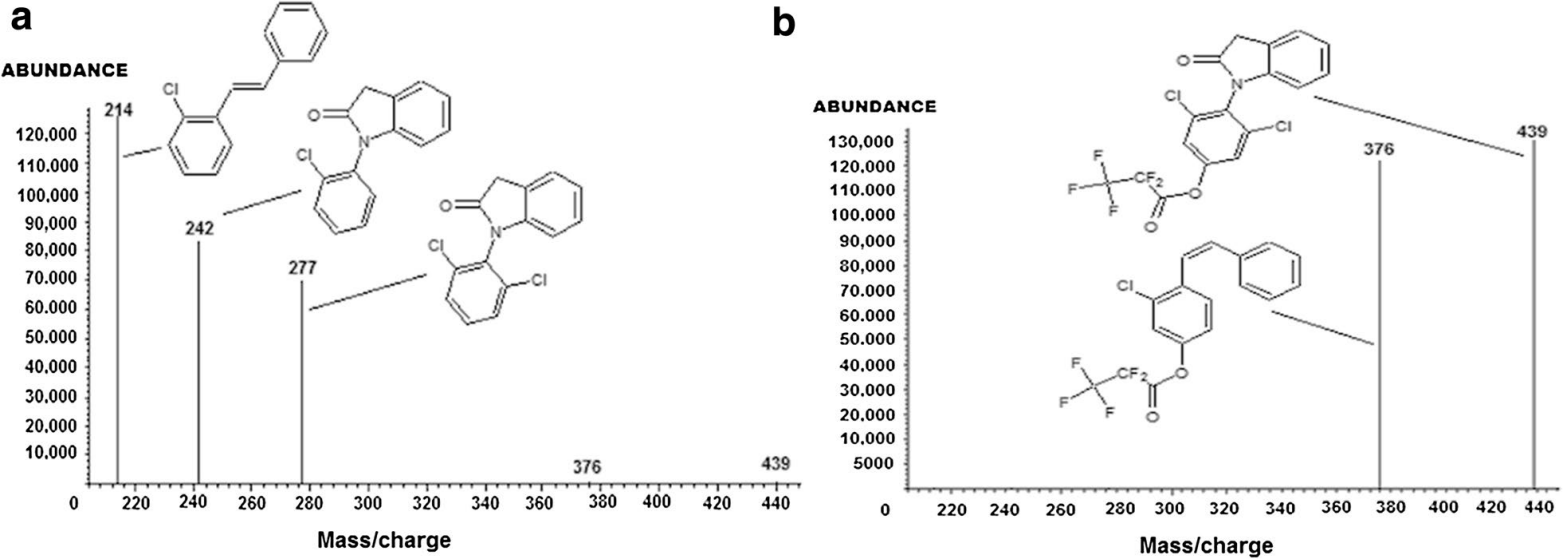
**a** Mass spectrum showing abundant ions for diclofenac derivative. **b** Mass spectrum showing abundant ions of 4-hydroxydiclofenac derivative

The mass spectra of diclofenac and the internal standard are shown in Fig. 3a, b. The derivatised indolinone ions for diclofenac and its internal standard fragment differently in the mass spectrometer giving rise to two distinctly different indolinone ions as shown in Fig. 3a, b.

### Linearity, sensitivity and specificity

During the validation study, calibration curves were generated over a diclofenac concentration range of 0.25–50 ng/mL. The method showed good sensitivity, specificity and linearity in the concentration range 0.25–50 ng/mL. The plots were linear over the concentration range 0.25–50 ng/mL.

The curves were all linear with a mean coefficient of determination of 0.9996, see Table 2. To evaluate the curve, the observed responses for the individual standards were substituted back into the equation in order to calculate the predicted concentrations based on the calibration curve. The limit of quantitation was 0.25 ng/mL. Using a signal-to noise ratio measure, the estimated limit of detection was 0.125 ng/mL.

**Fig. 4:**
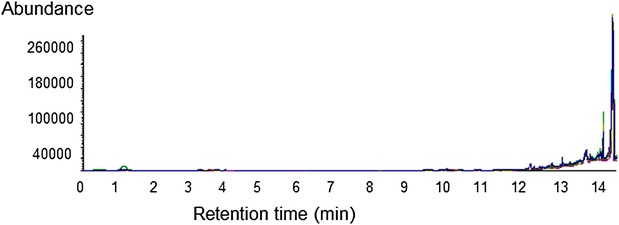
Overlay chromatograms of six blank plasma samples showing specificity

Furthermore, as can be seen from the Table 1, the percentage recovery of diclofenac in spiked plasma samples, was well within the accepted limit of 85–115 %, thereby showing no matrix effects. No notable peaks were seen in the region of interest when six blank plasma samples were analyzed, see Table 1. The retention time region of the chromatograph where diclofenac and 4-hydroxydiclofenac eluted was clear in these samples and demonstrated the specificity of the validated analytical procedure. No interference from endogenous compounds or metabolites of diclofenac was found around the elution times, however a matrix peak was observed at a different retention time see Fig. 4.

**Table 1:** Recovery of diclofenac standards when added to blank plasma showing no notable matrix effect (n = 6)

Nominal concentration (ng/mL)	Recovery Mean ± SD (%)
0.5	97.45 ± 6.21
1.1	98.63 ± 5.22
15	100.52 ± 4.73
40	97.59 ± 7.21

### Intra and Inter assay accuracy and precision

The inter-assay accuracy and precision were calculated from results obtained from quality control samples (N = 6) analysed at four concentrations (0.5, 1.1, 15 and 40 ng/mL of diclofenac in EDTA plasma representing LLOQ, QCL, QCM and QCH respectively) on three separate occasions, see Table 2.

**Table 2:** Summary of assay validation results including precision and accuracy data

Analyte (ng/mL)	QC (ng/mL)	Linear range (ng/mL)	LOD (ng/mL)	r^2^	Intra-day (N = 6)		Inter-day (N = 6)		Recovery %
					Precision % CV	Accuracy %	Precision, % CV	Accuracy %	
Diclofenac Na	0.5	0.25–50	0.125	0.9996	6.33	95.82	8.64	102.01	
	1.1				2.41	89.45	8.87	99.54	94.76
	15				5.39	91.12	7.70	99.47	91.77
	40				3.41	88.98	7.51	95.73	89.86

*LOD* limit of detection, *% CV* coefficient of variation

### Recovery

Our initial attempts gave a respectable recovery of the spiked drug at ca. 60 %. However, further experiments using acetone and sodium bicarbonate showed that the simple addition of these two reagents resulted in a dramatic increase in recovery by 50 %. Final recoveries were calculated during validation runs as shown in Table 2.

Intra and inter day precision (coefficient of variation) ranged between 2.41–6.33 and 7.51–8.87 % respectively, while intra and inter day accuracy ranged between 88.98–95.82 and 95.73–102.01 % respectively. The percent recovery of the three QC’s ranged between 89.86–94.76 %, see Table 2.

### Freezing and thawing cycles

The QCL = 1.1 ng/mL samples gave a mean result of 1.96, 2.02 and 1.88 ng/mL (n = 6) with the corresponding percentage change from freshly prepared samples of +9.49, +12.93 and +4.83 % for freezing and thawing cycles 1, 2 and 3 respectively. The QCM = 15 ng/mL samples gave a mean result of 18.85, 18.97  and 19.19 ng/mL (n = 6) with the corresponding percentage change from freshly prepared samples of +3.42, +4.11 and +5.29 % for freezing and thawing cycles 1, 2 and 3 respectively. The QCH = 40 ng/mL samples gave a mean result of 51.05, 51.23 and 50.85 ng/mL (n = 6) with the corresponding percentage change from freshly prepared samples of +4.32, +4.69 and +3.91 % for freezing and thawing cycles 1, 2 and 3 respectively. The data indicated that diclofenac was stable in EDTA plasma to at least three freezing and thawing cycles.

The validation results indicated that the proposed method is more efficient in detecting the non-steroidal anti-inflammatory drug diclofenac, in human plasma even at very low levels when only ca. 1000 µL of human plasma was processed. Under the extraction and chromatographic conditions employed, there were no detectable interferences by endogenous materials present in human plasma.

Three freezing and thawing cycles showed that diclofenac was stable in EDTA plasma. The average percent variation from freshly prepared EDTA samples, at three concentration levels, were 9.1, 4.27 and 4.3 % respectively.

Many GCMS derivatization reagents has been tried and tested in the past to get maximum sensitivity and ultimate recovery of diclofenac from human plasma. Choi et al. showed that when a mixture of PFPA and a mixture (1000:2:3, v/w/w) of *N*-methyl-*N*-trimethylsilyltrifluoroacetamide (MSTFA), ammonium iodide (NH_4_I), and dithioerythritol (DTE) were used as derivatisation reagents, the lower limit of quantification (LOQ) was 0.5 ng/mL. While we have used PFPA as a derivatisation reagent, with an improved LOQ of 0.25 ng/mL and a similar recovery to Choi’s work [32]. Yilmaz et al. described a method where MSTFA was used as derivatising agent (silylating reagent). Here, the LOQ was a factor of ten higher at 5 ng/mL with a recovery of about 96 % [31]. Others who have used PFPA as a derivatising agent include Borenstein et al. who achieved a lower limit of quantification (LOQ) of 1 ng/mL with a 95 % recovery and Kadowaki et al. who reported a LOD (LOQ not reported) of 0.2 ng/mL and recoveries of ca. 83 %. However, they used benzene as an extraction solvent which is more toxic [29]. Electro-membrane extraction (EME) and pulsed electro-membrane extraction (PEME) coupled with HPLC gave an LOD of 10 ng/mL an LOQ was not reported [25].

Our method has given a considerable improvement over the above methods with increased sensitivity LOQ 0.25 ng/mL and greater than 90 % recovery. Hexane was used in the sample preparations steps instead of heptane and benzene, as it is a relatively less toxic extraction solvent. Furthermore, use of hexane resulted in a higher recovery of >90 % as compared to the published lower recoveries (around 83 %) for heptane and benzene [16–20].

In short the developed and validated GCMS method for diclofenac satisfy all the criteria for US-FDA’s “Guidance for Industry Bioanalytical Method Validation:” [34] The method is very reliable and robust for quantitative determination of diclofenac in human plasma.

### Assay application

**Fig. 5:**
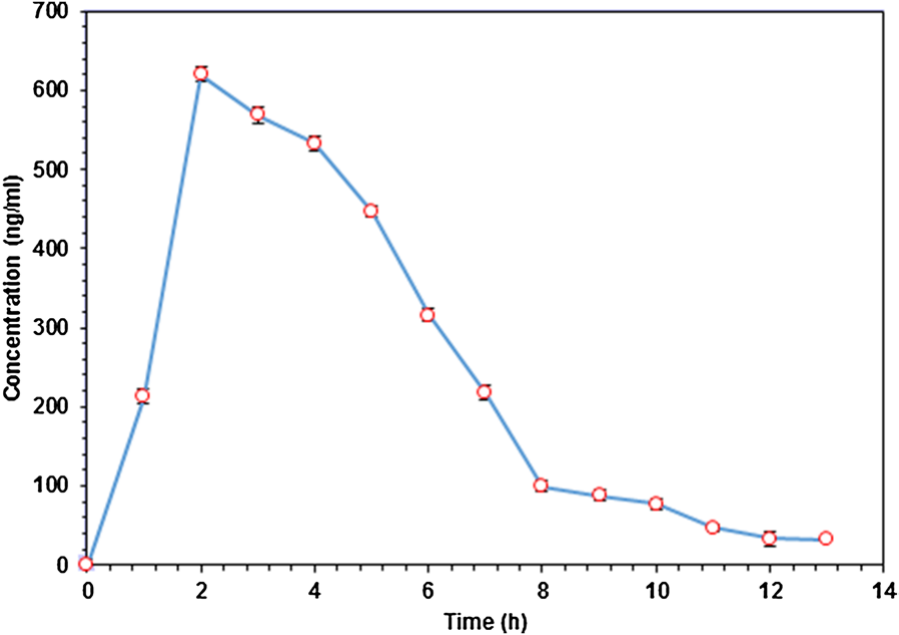
Plasma concentration–time profiles of Diclofenac following a single oral dose of 100 mg of modified release capsules (Rhumalgan XL). The *error bars* represent the standard error of mean

The pharmacokinetic study was conducted and applied to 30 volunteers who had been given an oral dosage of 100 mg of diclofenac sodium (Rhumalgan XL 100 mg modified-release capsules). The amount of diclofenac was determined between 0 and 12 h in human plasma. The mean plasma concentration–time curve is shown in Fig. 5.

Diclofenac sodium is rapidly absorbed from the gut and undergoes first-pass metabolism [17, 36]. Rhumalgan XL 100™ capsules give the peak plasma concentrations (C_max_) at approximately 2.1 h (T_max_), where T_max_ is the maximum time at which C_max_ was observed after administration. The total drug exposure, which is the area under the curve (AUC) over time was calculated from the concentration time data.

According to FDA guidelines, for generic drugs studies, the area under the curve (AUC) was calculated by the Linear Trapezoidal method, by applying, non-compartmental data analysis using the PK Solver 2.0 software (as an Excel add-on).

Here AUC_0–∞_ is the extrapolated value of the AUC curve to infinite time and AUC_0–12_ is the AUC time-concentration curve to the last measurable concentration at the 12 h time-point. The mean values of pharmacokinetic parameters estimated are shown in Table 3 [1–5]. Based on our new GCMS method, drug quality parameters like bioavailability and bioequivalence could be estimated accurately based on pharmacokinetic measures such as AUC and C_max_ that are reflective of systemic exposure. In humans, the pharmacokinetics of diclofenac retention and absorption show that it has high inter- and intra-subject variability [3, 5–7, 9–16]. In light of these previously reported inter- and intra- variability, our method (although assayed on a relatively small sample of 30 subjects) seems to be especially valuable as it showed very small variability and high reproducibility.

**Table 3:** Pharmacokinetic parameters, where AUC shows area under curve and C_max_ shows the peak plasma concentration of the drug after administration, T_max_ shows time to reach C_max_

Parameter	Mean ± SD	Confidence level (95 %)
C_max_ (ng/mL)	625 ± 8.4	13.4
T_max_ (h)	2.0 ± 0.45	0.72
AUC _0–12_ (ng/mL h)	3243 ± 9.8	15.6
AUC _0–∞_ (ng/mL h)	3331 ± 8.3	13.1

*SD* standard deviation

A possible reason for this reduction in inter- and intra-individual variability as compared to other methods may be the use of new extraction solvents such as hexane along with phosphoric acid, acetone and sodium bicarbonate for increased deproteination and PFPA as a robust and efficient derivatising agent.

The newly developed and validated method could have far reaching impact in pharmacokinetic and bioequivalence studies of diclofenac sodium in human patients. The proposed method might be applied to other human and animal matrices in future studies for accurate quantitation of diclofenac. This new method will also be instrumental in any future drug studies to show bioequivalence between generic and innovator drug products.

## Conclusions

The developed and validated method for the determination of diclofenac in human plasma is rapid, sensitive, specific, reproducible and robust, and offers better sensitivity than previous methods. It utilizes hexane which is a relatively less toxic extraction solvent as compared to heptane and benzene, while phosphoric acid, acetone and sodium bicarbonate were used for increased deproteination. Due to the very small variability and high reproducibility this method has been proved to be suitable for use in pharmacokinetic studies of diclofenac in human plasma, which demonstrates the possible adequacy of this assay for clinical studies.
